# Supplementary material for: Predicting speed of progression of lens opacification after pars plana vitrectomy with silicone oil
Source: PLoS One. 2022 May 20;17(5):e0268377. doi: 10.1371/journal.pone.0268377 (PMC9122216; doi:10.1371/journal.pone.0268377)
Supplement: S1 File — (DOCX) [file pone.0268377.s001.docx]

# Predicting speed of progression of lens opacification after pars plana vitrectomy with silicone oil

Supplemental Content

Philipp Schindler MD^1^*^†^, Luca Mautone MD^1†^, Vasyl Druchkiv^2^, Toam Katz MD^1^, Martin Stephan Spitzer MD^1^, Christos Skevas MD^1^

^1^Department of Ophthalmology, University Medical Center Hamburg-Eppendorf, Hamburg, Germany

^2^Department of Research & Development, Clínica Baviera, Valencia, Spain

^†^These Authors contributed equally to the work

***Corresponding author:**

Dr. med. Philipp Schindler, FEBO

Email: p.schindler@uke.de (PS)

# Surgical technique

As it is standard in our clinic surgeries were performed under local or general anesthesia according to the preferences of the patient. The same surgical method was used in all patients (23gauge vitrectomy). Trocars were inserted in the inferotemporal, superotemporal, and superonasal quadrants 3.0–4 mm posterior to the limbus. All eyes underwent a complete vitrectomy (scleral depression performed in all patients), followed by fluid-air exchange and endolaser treatment to re-attach and fixate the retina. At the end of the surgery siliconoil (2000cs or 5000cs) instillation was performed. After that microcannulas were removed from the eye. No sutures were needed to close the scleral or conjunctival openings. No intraoperative complications or lens touch occurred in any eye. None of the cases exceeded a surgery-time of 80 minutes.

**Study vs. fellow eye**

We saw that by applying variable selection method in predicting individual trajectories of the fellow eye no effect of time was detected. To compare study eye and fellow eye, we fit the model from the study eye on the fellow eyes.

**S1 Fig. Measured mean LD of study eyes vs. fellow eyes over time.**


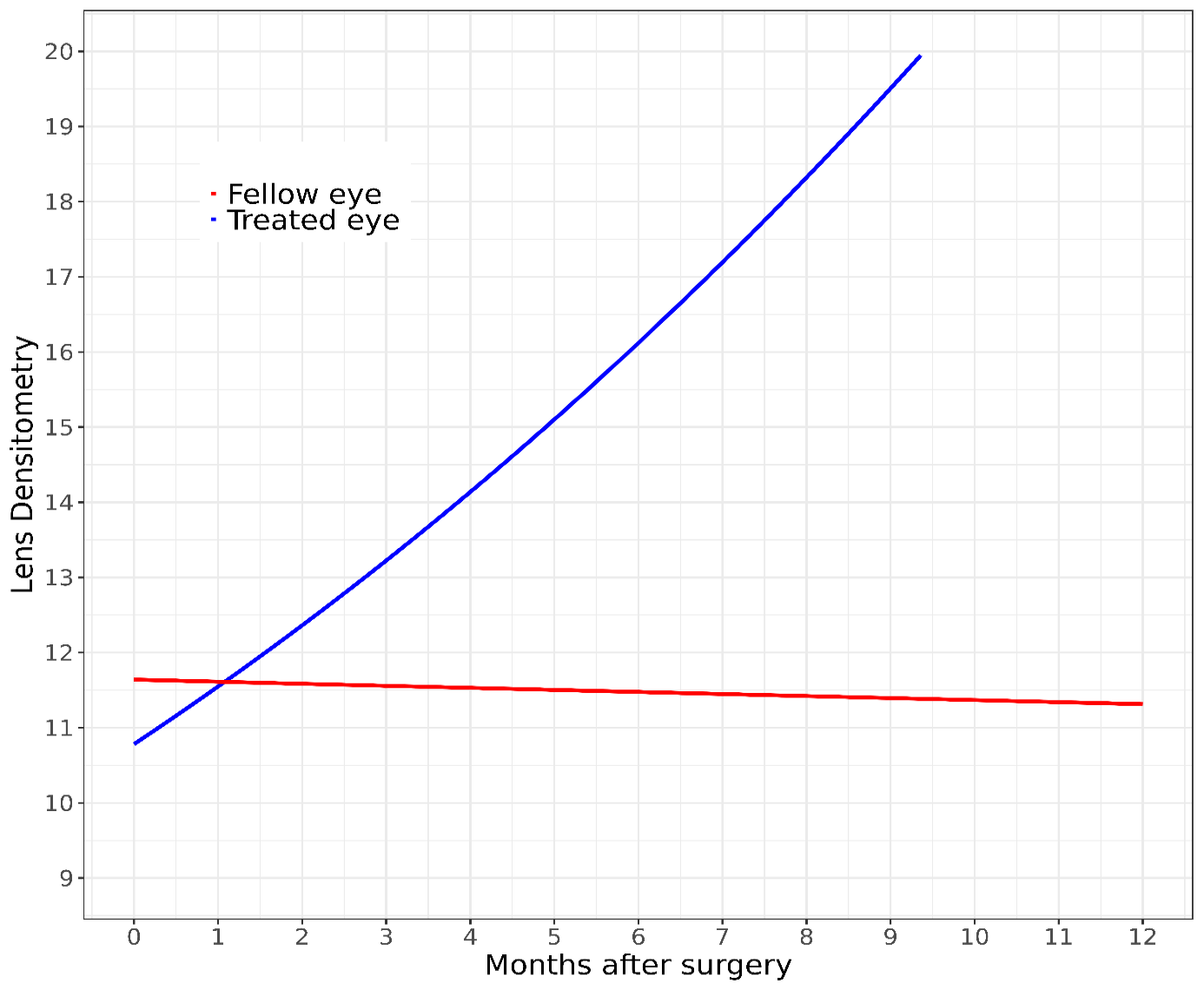


**S2 Fig. Individual trajectories of measured LD for fellow eyes.**


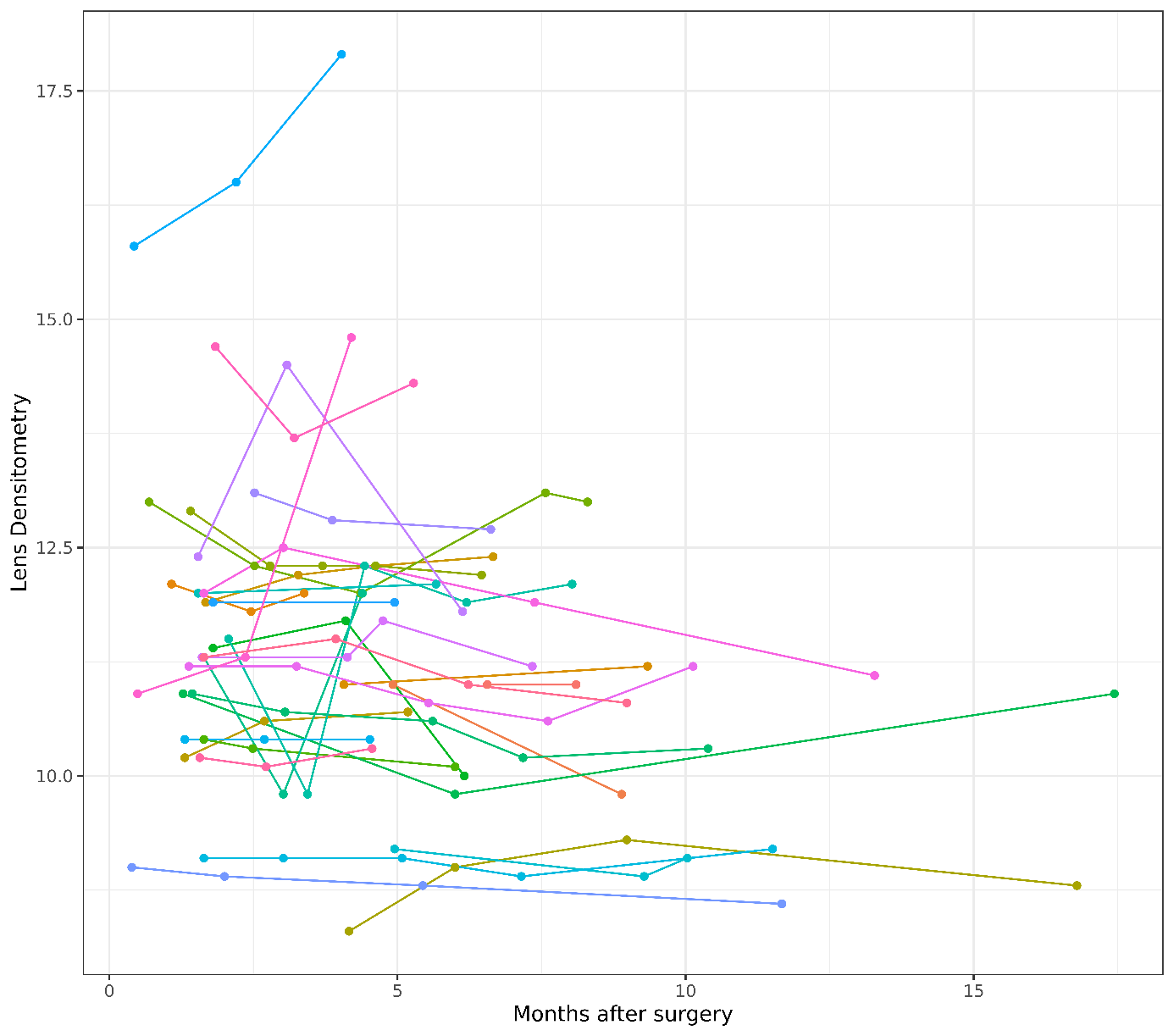


Results of the mixed effects regression model for fellow eyes is shown in S1 Table.

**S1 Table. Mixed effects regression model best fit for fellow eyes.**

|  | Dependent variable:  $\frac{\frac{\boldsymbol{LD}}{\boldsymbol{100}}}{\boldsymbol{1-}\frac{\boldsymbol{LD}}{\boldsymbol{100}}}$ |
| --- | --- |
| β_1_ Age | 0.026* (0.013) |
| β_2_ Baseline lens densitometry | 0.219*** (0.063) |
| β_3_ Age x Baseline lens densitometry | -0.003** (0.001) |
| β_0_ Constant | -4.269*** (0.620) |
| Observations | 84 |
| Log Likelihood | 95.199 |
| Akaike Inf. Crit. | -178.398 |
| Bayesian Inf. Crit. | -163.813 |

P-Values are given symbolically with asterisks: *p<0.1; **p<0.05; ***p<0.01. In parenthesis are standard errors of the coefficients.

Only age and baseline lens densitometry seem to be significantly related to LD, which we of course expect, since the baseline LD increases with increasing age. We see that time doesn't even enter significantly the model. Since time is not related to the trajectories the prediction would be the horizontal line that only goes up or down depending on baseline density and age.

# Model accuracy and detailed list of previously selected individuals for testing model performance

The deviation of observed and predicted data stretched from 0.27% to 2.42% (average 1.06%). (S2 Table)

The closer deviation reaches zero percent the more accurate is the prediction. The highest accuracy was achieved for ID 3 (63 years) with a high baseline LD of 13.4%. The worst accuracy was measured for ID 5 (age=59; baseline LD 13.2%). But at month 13.28 after surgery predicted LD for ID 5 was still very precise.

**S2 Table. Previously selected five individuals and related data of observed and predicted lens densitometries**

| ID | Age | Baseline  LD in % | Month after surgery | Observed  LD in % | Predicted  LD in % | Mean Deviation (%)  $\frac{\mathbf{Difference: Obs.-Pred.}}{\mathbf{number of measurements}}$ |
| --- | --- | --- | --- | --- | --- | --- |
| 1 | 54 | 10,0 |  |  |  | **0,67** |
| 1 |  |  | 1.64 | 12.2 | 11.5 |  |
| 1 |  |  | 3.02 | 12.5 | 12.8 |  |
| 1 |  |  | 4.39 | 15.8 | 14.1 |  |
| 2 | 59 | 11,7 |  |  |  | **1,00** |
| 2 |  |  | 2.07 | 12.6 | 12.6 |  |
| 2 |  |  | 3.44 | 11.4 | 13.9 |  |
| 2 |  |  | 4.43 | 15.6 | 14.8 |  |
| 2 |  |  | 6.20 | 17.5 | 16.7 |  |
| 2 |  |  | 8.03 | 17.9 | 18.8 |  |
| 3 | 63 | 13,4 |  |  |  | **0,27** |
| 3 |  |  | 2.52 | 13.4 | 13.0 |  |
| 3 |  |  | 3.87 | 13.6 | 13.8 |  |
| 3 |  |  | 6.62 | 16.0 | 15.8 |  |
| 4 | 63 | 11,8 |  |  |  | **0,95** |
| 4 |  |  | 1.61 | 11.0 | 12.0 |  |
| 4 |  |  | 4.13 | 13.4 | 14.3 |  |
| 4 |  |  | 4.75 | 14.3 | 14.9 |  |
| 4 |  |  | 7.34 | 16.4 | 17.7 |  |
| 5 | 59 | 13,2 |  |  |  | **2,42** |
| 5 |  |  | 1.64 | 15.5 | 12.8 |  |
| 5 |  |  | 3.02 | 18.3 | 13.8 |  |
| 5 |  |  | 7.38 | 22.4 | 17.6 |  |
| 5 |  |  | 13.28 | 23.7 | 23.8 |  |
|  |  |  |  |  |  | **average = 1,06** |
