# Supplementary material for: Predicting speed of progression of lens opacification after pars plana vitrectomy with silicone oil
Source: PLoS One. 2022 May 20;17(5):e0268377. doi: 10.1371/journal.pone.0268377 (PMC9122216; doi:10.1371/journal.pone.0268377)
Supplement: Attachment — Submitted filename: Response to reviewers.docx [file pone.0268377.s002.docx]

Hamburg, 11/March/2022

PLOS ONE®

**Response to reviewers**

Dear Reviewers,

First of all we want to thank you very much for your constructive criticism. We appreciate your comments and hope that we have answered your questions sufficiently clearly and satisfactorily.

Following you will find a list of your remarks and our corresponding answers.

Reviewer #1:

Answer: Our plan is to spread the statistical model among other surgeons with access to PentacamHR PNS measurements. When they plan to perform PPV without combined cataract surgery, we ask them to perform a PNS scan before surgery. Predictions by our model can be performed for any timepoint, for example one year after surgery. This can be made online on a freely accessible platform. After one year PNS scans should be repeated and compared to the calculated predictions.

Reviewer #2 remark 1): The authors have recently published a similar study, regarding C3F8 as opposed to silicone oil, which was indeed referenced in this study. It is hard to understand why the results of both studies were published separately. This causes a significant extent of this study to be replicated from the previous one. The authors should elaborate on this issue, as methods, results, and conclusions are also very similar.

Answer: Yes, statistical methods are the same. Dividing sample in training and test data sets -> mixed regression modelling with backward variable selection -> applying the resulted model on the test data set.

What essentially differs is the data set: In previous study the model is derived based on patients treated with gas and in the current study are patients treated with oil.

Applying same methodology on different data may produce different result. As you can see the derived models are slightly different. In model for patients treated with C3F8-gas the polynomial term for month is retained. And in the model for silicone oil there is no polynomial for month but there is significant higher order interaction: Months X Age X Baseline_LD

Literature and clinical observations showed that silicone oil is thought to have different impact on lens opacification than C3F8 gas. It was our goal to get reliable data for patients treated with oil vs. patients treated with C3F8 gas. Developing one mathematical model from a dataset containing both groups, oil and gas, would impede the differentiation of their effect. The aim of both studies is to describe progression of lens opacification with objective parameter as precise as possible and from that data develop a statistical tool that adds clinical relevance for the understanding of cataract progression and patient consultation for both types of endotamponade.

Reviewer #2 remark 2) and 3):

Answer: Please see lines 340-352 in the revised manuscript with track changes

Reviewer #2 remark 4): Can the authors provide information regarding the Variance inflation factors (VIFs) in the model?

Answer: It is not clear what you are implying. But, as expected, the VIFs for the parameters in the final model are very large. We should emphasize AS EXPECTED because we use interaction terms in our modelling. In models with interactions the large VIFs are not a problem and are expected. Furthermore the goal of the study is to make a prediction about densitometry trajectories and therefore we are interested much more in the parameter estimates than in their standard errors. Recall that no matter how great the multicollinearity among a set of variables is, it in no way compromises the estimates associated with the other variables in the regression. Nevertheless we are willing to give you some idea about the correlation among the three main predictors in the model: age, month and baseline densitometry. If we estimate the model with only these free parameters without interaction among them we derive following Variance Inflation Factors: VIFmonth=1.005, VIFage=1.62 and VIFbaseline_density=1.61. These VIFs are fairly low to cause any

concern.

Reviewer #2 remark 5): It would be best to add a citation to the sentence in lines 257-259.

Answer: We are not sure which sentences of the manuscript you are referring to, since line 257-259 already have several citations [21]–[24] and [19], [23], [25]–[28]. For a better aspect we added paragraphs.

Reviewer #2 remark 6): The authors should provide a reasonable explanation as to the major difference between the outcomes of different Lens densitometry (LD) baselines (10% vs 11%)? Could this be affected by other Confounding variables? Sample size?

Answer: Please see lines 298-314 in the revised manuscript with track changes. We rephrased this parts for a better clarification.

Reviewer #2: The authors mention exclusion criteria …How many patients did this entail? Was this a fair amount that represents the cohort? What was the reasoning behind their exclusion criteria? The authors should also provide an explanation as to why Diabetes Mellitus was not excluded, as opposed to other conditions.

Answer: In our clinic we approximately perform 1000 vitrectomies every year. The number of patients presenting to our clinic with primary rhegmatogenous retinal detachment varies between 300-400 cases per year. These patients usually come as emergencies. Among those patients the majority is already pseudophakic. Remaining phakic cases (on both eyes) receive Pentacam PNS and IOL-Master measurements within our routine diagnostic procedure independent of whether PPV is combined with cataract surgery or not. This ensures that surgery will be able to start as soon as possible without any further delay due to missing diagnostic examinations. When the surgeon has made his or her decision which procedure will be performed, we screen those cases for inclusion. So, the clinical decision for or against phakocvitrectomy was made completely independent of this study. About 10 to 15% of the cases that would have met the criteria were lost because these cases arrived at the weekend and no PNS diagnostics were available. Included patients received verbal information about the upcoming measurements including Pentacam PNS during their routine post-operative visits. An exact number of excluded patients cannot be given.

Diabetes mellitus (DM) was not excluded because of two reasons: First, DM is a frequently occurring condition in Europe and excluding those cases without any sings of diabetic retinopathy would lead to an even smaller cohort. Number two, diabetes is a known risk factor for cataract formation indeed, but if visually impairing cataract was present at the time of the first presentation of the patient, he or she would most likely have had combined phakovitrectomy. In addition, a recent meta-analysis showed that DM might not lead to nuclear sclerotic cataract (Li L, Wan XH, Zhao GH. Meta-analysis of the risk of cataract in type 2 diabetes. BMC Ophthalmol. 2014 Jul 24;14:94. doi: 10.1186/1471-2415-14-94. PMID: 25060855; PMCID: PMC4113025.) And some recent studies showed that DM could probably have some protective effect against cataract formation in vitrectomized eyes.

Reviewer #2 remark 8) and 9)

Answer: Please see lines 89-92 in the revised manuscript with track changes

Reviewer #2 remark 10): When giving the BCVA, could the authors also add the Snellen 20/X equivalents of the logMAR visual acuities listed?

Answer: units have been added in Table 1

Reviewer #2 remark 11) : Given the broad nature of readership of PLoS One, I think a supportive citation should be added.

Answer: We added a further citation for lines 340-343

Reviewer #2 remark 12) and 13):

Answer: Please see lines 344-346 and 350-359 in the revised manuscript with track changes

Reviewer #2 remark 14): Are there any data about the predictive value of other measures in the literature? It would be valuable to mention that in the discussion and provide relevant information, to get a sense of how well it does overall in comparison to other, perhaps more widely available, methods/instruments.

Answer: To our knowledge this and the predecessor study with C3F8-gas are the first studies that try to depict objective parameters for changes in lens status after PPV and, additionally, derive a mathematical model from that data to make future predictions for individual cases. Other models exist using artificial intelligence and deep learning to predict a risk for cataract from epidemiological data or to calculate the acuity of refractive power after cataract-surgery from preoperative measures.

Reviewer #2 remark 15) Due to the aforementioned, I think the conclusions drawn, especially in the way they were phrased – are too conclusive. It would best to use more cautious terminology.

Answer: We rephrased our conclusions according to the reviewer’s recommendations.

We want to thank you for your efforts.

Yours sincerely,

Dr. med. Phillip Schindler

Specialist for Ophthalmology

University Medical Center Hamburg-Eppendorf
